# How to become an expert educator: a qualitative study on the view of health professionals with experience in patient education

**DOI:** 10.1186/s12909-015-0370-x

**Published:** 2015-05-13

**Authors:** Margrét Hrönn Svavarsdóttir, Árún K. Sigurðardóttir, Aslak Steinsbekk

**Affiliations:** 1 Department of Public Health and General Practice, Norwegian University of Science and Technology, Trondheim, Norway; 2 St. Olavs Hospital, Trondheim University Hospital, Trondheim, Norway; 3 School of Health Sciences, University of Akureyri, Akureyri, Iceland

**Keywords:** Clinical competence, Professional competence, Coronary disease, Continuing education, Health educators, Health personnel, Patient education as topic, Secondary prevention, Qualitative research

## Abstract

**Background:**

Health professionals with the level of competency necessary to provide high-quality patient education are central to meeting patients’ needs. However, research on how competencies in patient education should be developed and health professionals trained in them, is lacking. The aim of this study was to investigate the characteristics of an expert educator according to health professionals experienced in patient education for patients with coronary heart disease, and their views on how to become an expert educator.

**Methods:**

This descriptive qualitative study was conducted through individual interviews with health professionals experienced in patient education in cardiac care. Participants were recruited from cardiac care units and by using a snowball sampling technique. The interviews were audiotaped and transcribed verbatim. The data were analyzed with thematic approaches, using systematic text condensation.

**Results:**

Nineteen Icelandic and Norwegian registered nurses, physiotherapists, and cardiologists, who had worked in cardiac care for 12 years on average, participated in the study. Being sensitive to the patient’s interests and learning needs, and possessing the ability to tailor the education to each patient’s needs and context of the situation was described as the hallmarks of an expert educator. To become an expert educator, motivation and active participation of the novice educator and a supportive learning environment were considered prerequisites. Supportive educational resources, observation and experiential training, and guidance from experienced educators were given as examples of resources that enhance competence development. Experienced educators expressed the need for peer support, inter-professional cooperation, and mentoring to further develop their competency.

**Conclusions:**

Expert patient educators were described as those demonstrating sensitivity toward the patient’s learning needs and an ability to individualize the patient’s education. A supportive learning environment, inner motivation, and an awareness of the value of patient education were considered the main factors required to become an expert educator. The experienced educators expressed a need for continuing education and peer support.

## Background

Providing patient education can be challenging; it has become more complex in recent years due to aging populations [1, 2], cultural diversity [2], and decreased length of hospital stays [1]. Developments in society and health science [2] and, more recently, the use of social media in patient education [3] have placed a demand on educators to keep up to date with evidence-based medicine and the use of information technology. Patients request more information and participation in decisions concerning their health [4], and the move from the medical model to patient-centered care [5] requires increased competence in communication skills. Finally, lifestyle changes emphasized in secondary prevention indicate that health professionals need specific training in communication and lifestyle counseling [6].

As the leading cause of death and disability in Europe [7], coronary heart disease (CHD) is associated with an unhealthy lifestyle. The beneficial effect of lifestyle changes and adherence to recommended treatment on CHD mortality and morbidity has consistently been confirmed [6, 8, 9].

Patient education has been defined as, “Any set of planned, educational activities designed to improve patients’ health behaviors, health status, or both” [10]. As a facilitator of lifestyle change and risk factor reduction [11, 12], patient education is a core component in secondary prevention of CHD. In addition, patient education results in higher perceived control over the disease [13] and possible beneficial effects on health-related quality of life [14].

Health professionals skilled in educational science and lifestyle counseling are essential for secondary prevention [15]. Continuing education for health professionals can improve professional practices and healthcare outcomes for the patient [16]. However, there are concerns about the limited opportunities for continuing education focusing on patient education [17, 18]. The lack of emphasis on educational and behavioral science in cardiovascular educational programs is apparent in the literature [19, 20], and the need to develop continuing education for health professionals has been recognized [15, 21, 22].

Characteristics of expert nurses have previously been described in the literature [23]. However, to our knowledge, factors that enhance the development of an expert educator have yet to be studied. Our previously published study discussed the knowledge and skills needed for patient education [24]. In this study, we highlight resources and activities required for enhancing competence development in patient education.

The aim of this study was to investigate the characteristics of an expert educator according to health professionals experienced in patient education for patients with CHD, and their views on how to become an expert educator.

## Methods

This descriptive qualitative study used semi-structured face-to-face individual interviews to collect data. This design was chosen as an appropriate method of data collection related to personal perspectives and beliefs [25].

### Participants

The aim was to recruit health professionals in Norway and Iceland who possess experience in providing patient education to individuals with CHD. To recruit the participants, the first author introduced the study to health professionals working in cardiac care units. The first participants were asked to recommend other possible participants (snowball sampling), who were then chosen purposefully to ensure variation in age, gender, profession, work experience, and experience in patient education.

### Data collection

Data were collected between April and August 2013. The interviews were conducted by the first author in the participants’ native language (Icelandic or Norwegian) at a location chosen by the participants. The interviews were audiotaped and transcribed verbatim. The average interview duration was 40 minutes (range 23–64 minutes).

The main question asked in the interviews was, “What do you consider the optimal training in patient education for inexperienced educators who provide education for adults recently diagnosed with CHD?” The participants were additionally asked to describe their own learning needs and describe their ideas of an expert educator for individuals with CHD. The participants were informed that patient education was understood to cover a very broad range of individual- and group-based formal patient education, information giving, support, and lifestyle counseling.

### Ethical considerations

The study was conducted in accordance with the Declaration of Helsinki. The study was not subject to approval of a Research Ethics Committee as no sensitive or personal health information was collected [26, 27]. Participants were provided with written and oral information about the study and informed that they could withdraw at any time. Written informed consent was obtained from the participants before the interviews were conducted. Confidentiality was assured by keeping the audio files locked down and de-identifying the transcripts; the data were only accessible to the authors.

### Analysis

The data were analyzed after each interview, using a thematic approach based on Malterud’s systematic text condensation [28]. The analyses started by reading the transcribed interviews to obtain a general impression and identify the preliminary themes. Next, the transcriptions were systematically reviewed line by line to identify meaning units, which were then classified and sorted into themes. The third step involved sorting the meaning units within each theme into subgroups and reducing the content to a distillation of rephrased quotations, maintaining as much of the original terminology used by the participants as possible. Finally, the contents of each code group were summarized in generalized descriptions and concepts. Interviews were conducted until no new themes emerged from the analyses.

The analyses were performed by the first author who has experience in providing patient education to individuals with CHD. To avoid preconceptions affecting the reflexivity of the results, the interview guide and the interpretation of the interviews were critically discussed between the co-authors and with a team of experienced researchers. The analysis was validated by a thorough review of the original transcript of each interview to ensure all points of significance were reflected in the results. The Icelandic and Norwegian citations were translated into English by the first author, who is competent in these languages, and validated by co-authors. The citations that best illustrated the themes were chosen to support the results and reflect the multi-professional diverseness. Citations are marked with the participant’s profession and self-evaluated experience in patient education.

## Results

Nineteen Icelandic and Norwegian health professionals were interviewed (Table 1). Their mean length of clinical experience in cardiac care was 12 years (range 0–32 years). All participants had experience of in-hospital patient education, and 18 had experience in patient education after discharge from hospital. Six of the participants had experience of counseling in nurse-led clinics. Five nurses were specialists in cardiology, and one of the physiotherapists specialized in cardiopulmonary rehabilitation. Both physicians were cardiologists.

**Table 1:** Demographic characteristics of the participants

	Number
Gender	
Female	17
Male	2
Age	
25–39	7
40–49	9
50–62	3
Nationality	
Norwegian	11
Icelandic	8
Profession	
Registered nurse	14
Physiotherapist	3
Cardiologist	2
Highest academic degree	
BSc	13
MSc	4
PhD	2
Source of competence in patient education	
Self-study (e.g. books/literature)	17
Supervision by an experienced colleague	14
Undergraduate education	12
Postgraduate education	12
Patient education course	7
Experience in patient education	
>3 years	14
1–3 years	3
<1 year	2
Self-evaluated experience in patient education	
Little experience	0
Average experience	3
Experienced	13
Extensive experience	3

The participants described the development from novice to expert in different ways. However, the development was commonly seen as a process that develops over time, through education, long-term clinical experience in cardiac care, a supportive learning environment, and personal motivation.

The findings were categorized into eight themes. The first two themes present the characteristics of expert and novice educators. The next two themes indicate the inner motivation and engagement in patient education, which is fueled by a supportive learning environment and peer support. The last four themes present concrete actions that can enhance the developmental process of the expert educator including the use of resources such as standard instructions and educational material, observation and experiential training, and mentoring and guidance from expert educators. The participants’ suggestions for resources and activities to enhance competence development all had a clear focus on individualization and evidence-based patient education. See subsection for resources and activities for competence development in patient education.

**Table:** 

**Resources and activities for competence development in patient education**
To be active in knowledge seeking and own training in patient education. To spend time reflecting and evaluating own performance. To have the opportunity to ask and receive answers to questions. To have dedicated time for theoretical learning and updates on new developments. To attend basic and advanced educational courses and conferences. To receive training from a mentor or experienced educator. To have access to peer support and role models. To have access to forums for knowledge sharing, discussions and consultations. To get guidance on literature searches and selecting patient educational material. To have access to a central collection of literature and research articles. To have access to clinical guidelines, instructions and checklists. To have access to standardized patient educational material and educational sessions. To have access to technical assistance while preparing and implementing patient education. To participate in training through case studies, roleplaying, group work, and discussions. To observe patient education in various settings from experts in patient education. To rehearse educational sessions under guidance. To get guidance in preparing, evaluating, and individualizing the educational session. To conduct patient education under supervision. To receive constructive critical reflection on own performance in patient education. To participate in the development of patient educational programs and educational material.

### Characteristics of expert educators

An expert patient educator was described as a health professional with advanced, up-to-date theoretical knowledge in cardiology and educational science, a holistic view of the patients’ situation, and sensitivity to and knowledge about their psychological well-being. Confidence and excellent communication skills were also seen as hallmarks of an expert educator, which included disseminating information in an interesting way that is clearly understood by the patient, and creating effective dialogue to motivate patients to perform necessary lifestyle changes. An experienced physiotherapist stated:

*“It’s a challenge for a professional with a wealth of knowledge to present it in a way that makes them* [the patients] *feel safe and confident to ask questions.”*

However, the most prominent signs of an expert educator were considered the ability to know when a patient is ready to receive information, being sensitive to the patients’ interests and learning needs, and being able to adjust the education to each patient’s needs and the context of the situation. An experienced cardiologist described an expert as:

*“That* [the expert] *is someone who knows when the patient is ready to receive information. You should know which information is beneficial for the patient. You should know how to disseminate the information and motivate the patient to receive the information. That is an expert.”*

### Characteristics of novice educators

A novice educator was described as having little clinical experience in cardiac care and patient education. Mainly due to this, the novice was said likely to exhibit underdeveloped communication skills and lack sensitivity toward the patient’s interests and needs, thus limiting the capability of the novice educator to prioritize information according to the patient’s needs. It was recognized that some novices have good theoretical knowledge and disseminate a wealth of good information, but they may not be capable of individualizing the educational session or selecting the most relevant information for the patient. A nurse with average experience described an example of novice educators’ capability:

*“I believe the new beginner, the novice, is in the present; he has enough to deal with. They see the patient here and now. I believe it takes several years before they can see the patient holistically, see his whole life, the consequences, and what may happen.”*

### Motivation and engagement

Several participants highlighted the necessity for a novice to have inner motivation and an ability to engage in order to become an expert educator. Awareness of the value of patient education and taking an active role in knowledge seeking and own training, were also deemed necessary. When describing why some health professionals become good educators while others do not, an experienced nurse said:

*“*[…] *because some lack interest. Even though they have long experience, they may not be interested in this* [patient education] *or not dedicated, while others are engaged from the beginning.”*

Some participants mentioned how interest in the patient helped motivate them to further their learning, how listening to the patient had helped them to discover which knowledge they lacked, and how they had learned from patients’ experiences and concerns. An experienced nurse stated:

*“When there are questions we don’t have the answer to, one needs to be undaunted in admitting it, and just say, ‘I will find out for you.’ You learn a lot that way.”*

### A supportive learning environment

A supportive learning environment at the workplace was considered motivational, inspiring knowledge seeking and facilitating competency development. Examples of factors considered to be motivational included having dedicated time at work for knowledge development, peer support, and informal and formal knowledge sharing. Some participants expressed an unfilled need for easy access to consultations and discussions with others, especially in difficult educational situations. They suggested multidisciplinary team meetings, networks of professionals in patient education, and conferences to enhance knowledge sharing. An experienced cardiologist said:

*“*[…] *this is the way I do it, how do you do it* [patient education]? *I have never had that conversation with another physician.”*

Many participants commented that novice educators need a significant amount of time to develop their knowledge and highlighted time constraints as a barrier to development. Some of the nurses claimed that owing to a lack of time at work, health professionals need motivation to study during their leisure time. However, not all participants were eager to participate in continuing education, as an experienced cardiologist explained:

*“I am terrified of everything that uses up my time. If you can participate in a single seminar, that is fine, but the days are so full of tasks. You should always aim at quality but this is about getting through your day.”*

### Supportive educational recourses

To counteract limited time and enhance learning, guidance in finding relevant literature and a central collection of literature and patient educational material was recommended. Standardized educational sessions, standard instructions, and clinical guidelines were reported as valuable sources of information, especially for novice educators, but they were also considered profitable for the expert educator. A nurse with average experience explained the advantages of such supportive educational resources:

*“You will be more confident in what you are doing, you get the courage to open up on issues with the patient and, with that, you gain competence.”*

Clinical guidelines were also considered a quality assurance that promote evidence-based patient education, as they could facilitate coordination of patient education and reduce the time needed to spend updating themselves. An experienced nurse explained:

*“They* [the clinical guidelines] *facilitate my work, you can organize your work better and be more focused in what you are doing.”*

Negative aspects of standard educational material were considered the potential risk of outdated material, since there may not be time to obtain the updates, and the difficulty adjusting the education to individual needs and contexts, particularly for the novice educator, who may be too fixed on the standard instructions.

### Building experience through observation and experiential training

The participants had mainly gained competence in patient education through experience, which they recognized as invaluable, and frequently stated the need for training in providing patient education and communicating with patients.

Some participants had observed novice educators trying to avoid providing patient education through fear of receiving unpredictable questions from patients or insecurity in a new situation, which they believed might come from not having tried it before. Their suggestion to overcome the situation was to encourage the novice educator to rehearse the educational session and gain secondary experience through observation of more experienced educators and experiential training.

The value of observing others was said to increase awareness of effective communication skills such as using appropriate language, learning how to explain and respond to questions, and getting an impression of what patient education entails. One of the experienced nurses commented:

*“It would be ideal if there were some instruction programs and a chance to observe a nurse providing patient education more than once, maybe two or three times, and then they would provide the education themselves with support* [from the nurse].”

Experiential training in the form of roleplaying and rehearsing the educational session were suggested not only to get experience but also to gain a better understanding of the motivation that the educator needs to evoke in the patient and to increase the educator’s consciousness of their communication skills and confidence in meeting patients. Another experienced nurse talked about the value of a more theoretical approach:

*“In communication training, you need to read and do exercises. Written exercises, I find them helpful. To have clinical examples, in which the patient says this, how do you respond? And you write down your answer according to this specific method, where the patient is a participant, who you are trying to motivate.”*

Roleplaying and rehearsing the educational situation could be implemented with colleagues serving as surrogate patients, using artificial patient case scenarios, or a scenario from the educator’s life. Although videotaping one’s own teaching was considered a good method, there was a concern that this could be threatening or uncomfortable for some.

### Moving from novice to expert educator

When asked about how to become an expert educator, the participants mainly described the need for experience, support, and supervision. Supervised practice either as informal guidance from different educators or, more preferably, a formal mentorship from an experienced educator. A structured mentoring program would allow the mentor to become aware of the novice educator’s process of learning, limits, and capabilities, making them better able to individualize the supervision. On the other hand, guidance from various educators would raise the possibility of learning a variety of educational strategies and methods. An experienced nurse explained:

*“To have access to someone who has more knowledge than you, has a lot of experience, is very important. Not only to receive knowledge but also to discuss problems that arise in individual interviews and in patient education, how you handle those situations.”*

Participants with experience in training other health professionals in patient education emphasized the importance of using constructive critical reflection and encouraging the novice educator to ask questions. This would enhance the learning process and adoption of good practices, and raise awareness of the pitfalls. An experienced nurse commented:

*“*[…] *and then I believe it is time to perform, but maybe under the supervision of the professional you learned from and get feedback, I believe that is extremely important, what did you do well and where can you improve.”*

However, some participants were concerned that the presence of an experienced educator in the educational setting could result in a passive role for the novice educator and, instead, suggested that supervision should focus on preparing novice educators for educational sessions especially regarding how to prioritize and adjust the patient education to individual patients’ needs.

Some participants described how the challenges an educator undertakes should increase in complexity, beginning with providing individual patient education, in which the novice educator has time to practice with only one patient, thus making it easier to observe and reflect on one’s actions. An experienced nurse stated:

*“The first step would be one-to-one, discussing the disease with the patient and initial education about the disease, lifestyle, and the proceedings.”*

Once confident, the educator should proceed to providing group patient education and facilitate discussions between patients. An experienced nurse explained the difference in challenges between individual and group patient education:

*“Several patients in the discussion group or group patient education are more demanding, because you need to moderate discussions and involve more patients. That is more challenging.”*

For individual counseling and follow-ups, longer experience and more extensive education and training were considered necessary, since this requires not only a broad knowledge of many areas but also the ability to motivate and help patients to adopt lifestyle changes unique to their situation.

### How to remain an expert educator

Those participants with lengthy experience in patient education and even responsibility in training and instructing health professionals in patient education expressed a need for additional continuing education for themselves to further improve their competency. When talking about his own learning needs, an experienced physician stated:

*“To have more training in communication, you know, to grasp what people have learned* [from the patient education] *and what they want to know. It is this individualization and communication.”*

In addition to the advice described in previous themes, the participants saw the need to examine their own performance, while focusing on their limitations and strengths. An experienced nurse explained about her learning needs:

*“What would be beneficial for someone who has already acquired a lot of knowledge and has long experience is some kind of training where your performance will be observed and evaluated,* […] *where you get feedback on what in your performance is working well and what is not.”*

One activity suggested by the participants was for expert educators to visit hospitals and clinics that lead the way in patient education, to receive introduction to educational programs and educational material and observe another expert educator providing patient education. Another suggested activity would enable the expert educator to design and implement an educational session and receive peer-evaluation, feedback, and instructions from a mentor. Although inexperienced with this form, several participants considered it the next step in their learning process.

## Discussion

The ability to know when a patient is ready to receive information, being sensitive to the patients’ interests and learning needs, and possessing the ability to adjust the education to each patient’s needs and the context of the situation were described as hallmarks of an expert educator. For developing from novice into an expert, inner motivation, active participation of the educator, and a supportive learning environment were considered prerequisites. Supportive educational resources, observations, and experiential training and guidance from experienced educators were suggested actions to enhance the developmental process of the expert educator. Experienced educators expressed the need for peer support and inter-professional cooperation to further develop their competency.

An expert patient educator was described in this study as a health professional with advanced, up-to-date theoretical knowledge in cardiology and educational science. Knowledge is part of clinical competence and includes using evidence-based current knowledge as well as an awareness of the need for knowledge and where to find it [29]. Possessing sufficient knowledge [30] and being professionally up to date are therefore considered crucial in developing competence [31]. The participants in this study were concerned that a lack of knowledge and confidence could add to health professionals’ reluctance to conduct patient education, thus hindering their professional development. Lack of knowledge has been identified as a barrier to the implementation of patient education [18] and a lack of resources, structured training, and skills development is considered a barrier to the implementation of CHD secondary prevention [22]. It is therefore concerning that in previous studies, nurses have reported their inactivity in reading literature related to patient education and failure to follow the development of knowledge in those areas [32]. Reluctance to conduct patient education and lack of knowledge in this area are issues that must clearly be addressed if health professionals are to improve their competencies in patient education.

A working environment of mutual respect, partnership, support, trust, and valued staff has been recognized in previous research as an inspiration to learn and develop [30, 33]. Time constraints and heavy workloads present obstacles to motivation for formal continuing education, at least for some of our participants. Professional development and learning through work depends on the employer’s support [31, 34]. Several nurses in this study stated that, in order to stay up to date on new developments, they needed to be motivated to study during their leisure time. This situation is supported by previous studies, which have shown that nurses use their leisure time for continuing education [22] and that managers expect them to do so [31]. This emphasizes the importance of considering health professionals’ preferences and motivation as well as their clinical reality and managerial support when designing continuing educational interventions.

Showing sensitivity to the patients’ interests and learning needs and individualizing patient education were considered hallmarks of an expert educator. The ability to meet patients’ individual needs has long been central to the role of an expert educator, as emphasized by Benner [35], who considered that capturing a patient’s readiness to learn and knowing when to move ahead were competencies of an expert and key aspects of effective patient education. In this study, novice educators were said to have a tendency to focus on specific tasks, rather than taking a holistic view of the patient, and they rely on standard instructions. Research has demonstrated that experts are superior to novices in recognizing patients’ cues and obtaining a total picture of the patient [36]; they do not rely on rules and guidelines but operate from an understanding of each patient’s situation [35].

Experience is considered a requisite for expertise [35] and is described as the most important factor in developing competence [33]. In this study, experience was considered invaluable in developing the skills that increase the ability to read the patients and meet them where they are. An active role and reflection of the health professional were considered necessary if experience was to result in expertise. Reflecting on experiences [23] and training in reflective thinking and relevant feedback [30] are important elements in developing competence [37]. This corroborates our findings, as the participants saw mentoring and constructive critical reflection on patient educational experiences and performance as important factors that enhance expert development.

In accordance with prior research [38], further training for experienced educators was deemed necessary to ensure high-quality patient education. When talking about the educational needs of experienced educators, many complained about scarce opportunities and wished for more peer support, inter-professional cooperation, and mentoring, indicating that experts’ learning needs are not being fulfilled. To facilitate more contact and discussions with other expert educators, a network of professionals in patient education was suggested. A lack of forums for joint reflection and discussions on difficult patient educational situations has previously been reported [37], and the need for regular forums for discussions of patient education has been suggested [32]. In this study peer support was the factor most frequently mentioned as an important motivating factor for competency development in patient education.

### Strengths and limitations

The main strength of this study lies in the long-term experience the majority of the participants had in patient education in cardiac care and that most had experience in various educational settings. In addition, some had experience in training health professionals in patient education, who therefore possessed a good understanding of the educational needs of both novice and experienced educators.

However, participants with less experience were in the minority, and there were no inexperienced participants. Therefore, including more participants with limited experience may have provided additional information about the educational needs of novice educators. The major limitation of the study was that the results were based on the views and professional opinions of health professionals and not on what they actually do. This approach was consciously chosen because of the absence of comprehensive descriptions of novice and expert educators for individuals with CHD and their educational needs. Even though the participants in this study worked within CHD care, the findings might be transferable to other settings, as they, in part, resonate well with what others have found. In addition, there were no apparent differences between the Icelandic and Norwegian participants.

## Conclusion

Having a holistic view of the patient, being sensitive to the patients learning needs, and having the ability to individualize patient education were considered essential competencies of an expert educator. Engagement and motivation in patient education along with awareness of the value of patient education and a supportive learning environment are prerequisites for becoming an expert educator. The experienced educators expressed a need for continuing education and support to further improve their competency. Structured training, peer support, and mentoring from experienced educators could increase the value of clinical experience, enhance the development of experts in patient education, and help to further develop the experts’ competencies
